# Supplementary material for: Surface protein glycosylation conserved in the human pathogen Mycoplasma genitalium and retained in the synthetic organism JCVI-Syn3A
Source: PLoS One. 2025 Sep 22;20(9):e0329506. doi: 10.1371/journal.pone.0329506 (PMC12453214; doi:10.1371/journal.pone.0329506)

Loading order for both gels (Fig 1):

1. Molecular weight marker (PageRuler ™ Plus prestained protein ladder)
2. *Mycoplasma mycoides* GM12 lysate
3. JCVI-Syn3A lysate
4. *Mycoplasma genitalium* G37 lysate

Coomassie stained gel (50 ms exposure time, imaged by with a BioRad Gel-Doc XR with white light box filter)

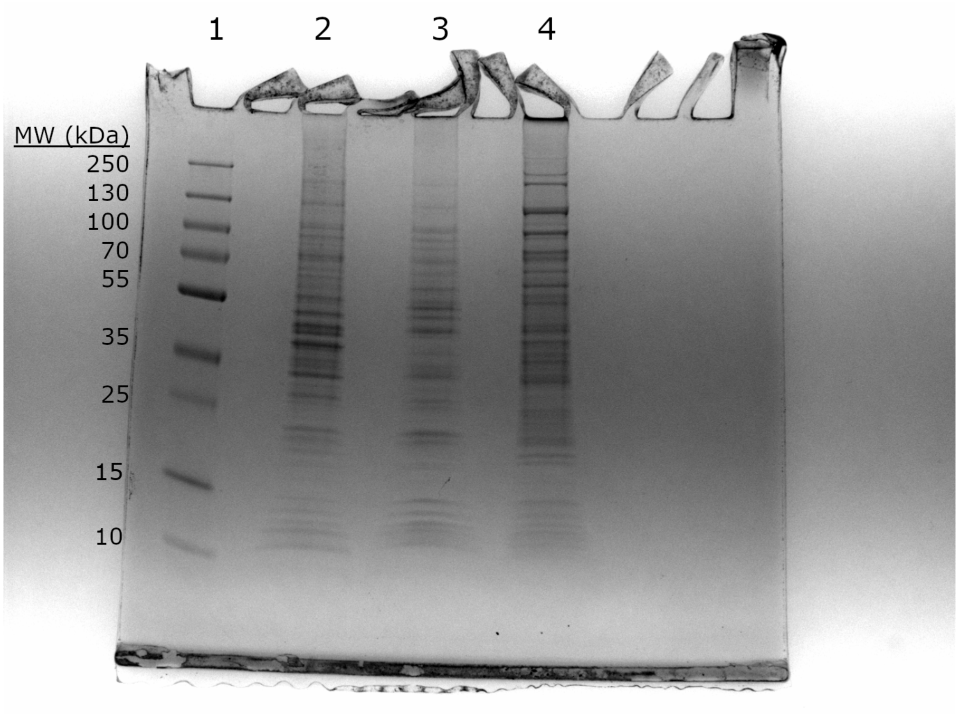

Pro-Q™ Emerald 300 glycostain gel (10 s exposure time, imaged by BioRad Gel-Doc XR)

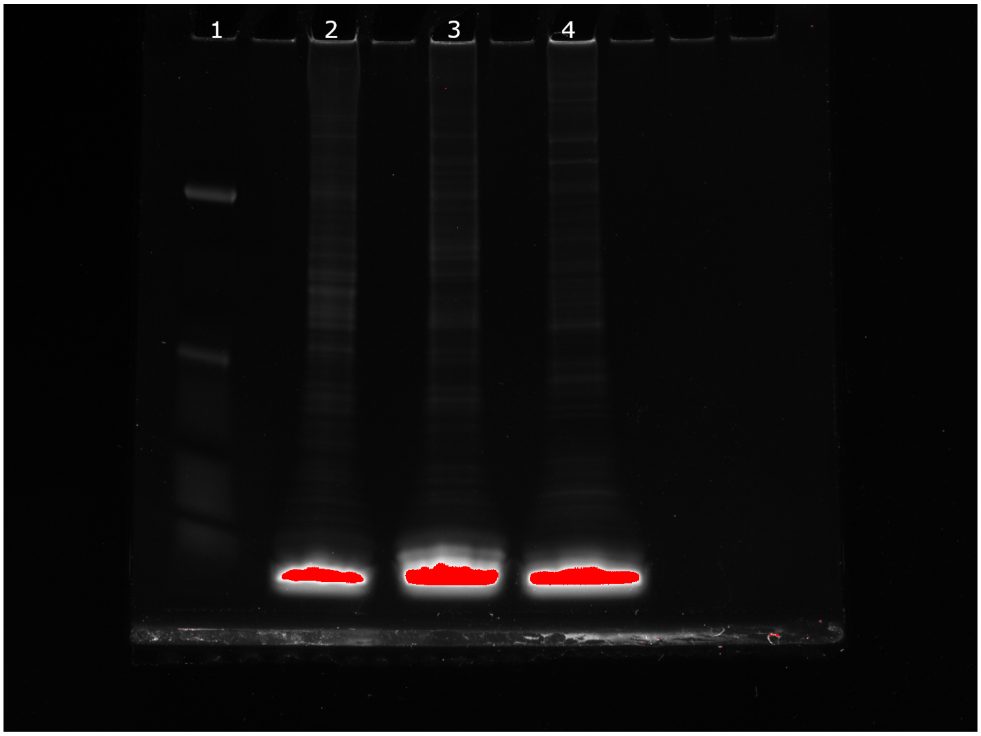

Supplement: S1 Fig — (PDF) [file pone.0329506.s001.pdf]
