# Supplementary material for: Surface protein glycosylation conserved in the human pathogen Mycoplasma genitalium and retained in the synthetic organism JCVI-Syn3A
Source: PLoS One. 2025 Sep 22;20(9):e0329506. doi: 10.1371/journal.pone.0329506 (PMC12453214; doi:10.1371/journal.pone.0329506)
Supplement: S2 Fig — (PDF) [file pone.0329506.s002.pdf]

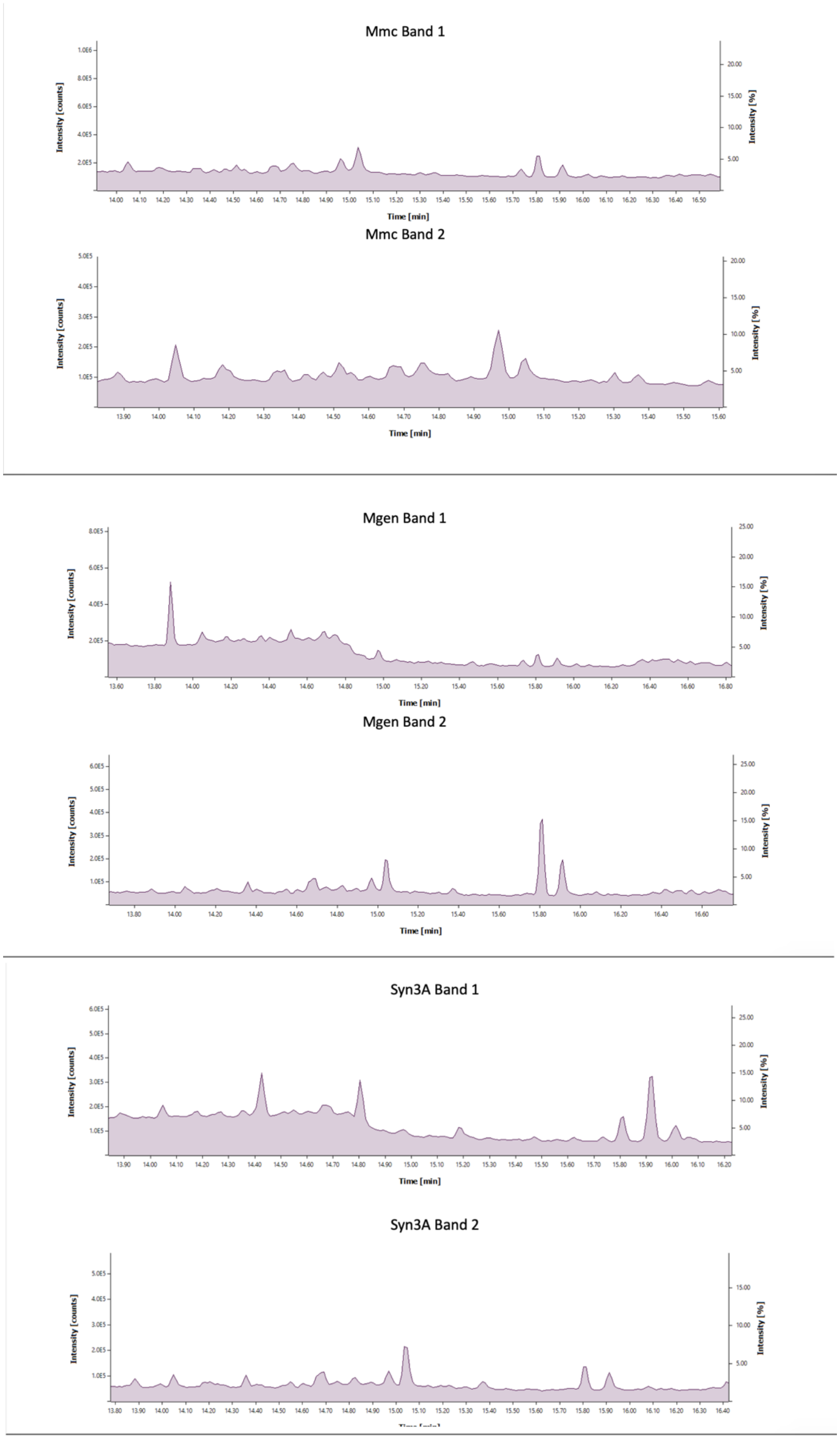

S2 Figure. Gas chromatograms from all excised bands in Fig 1 showing the presence of glucose and mannose in each sample.
