# Supplementary figures and images for: Surface protein glycosylation conserved in the human pathogen Mycoplasma genitalium and retained in the synthetic organism JCVI-Syn3A
Source: PLoS One. 2025 Sep 22;20(9):e0329506. doi: 10.1371/journal.pone.0329506 (PMC12453214; doi:10.1371/journal.pone.0329506)

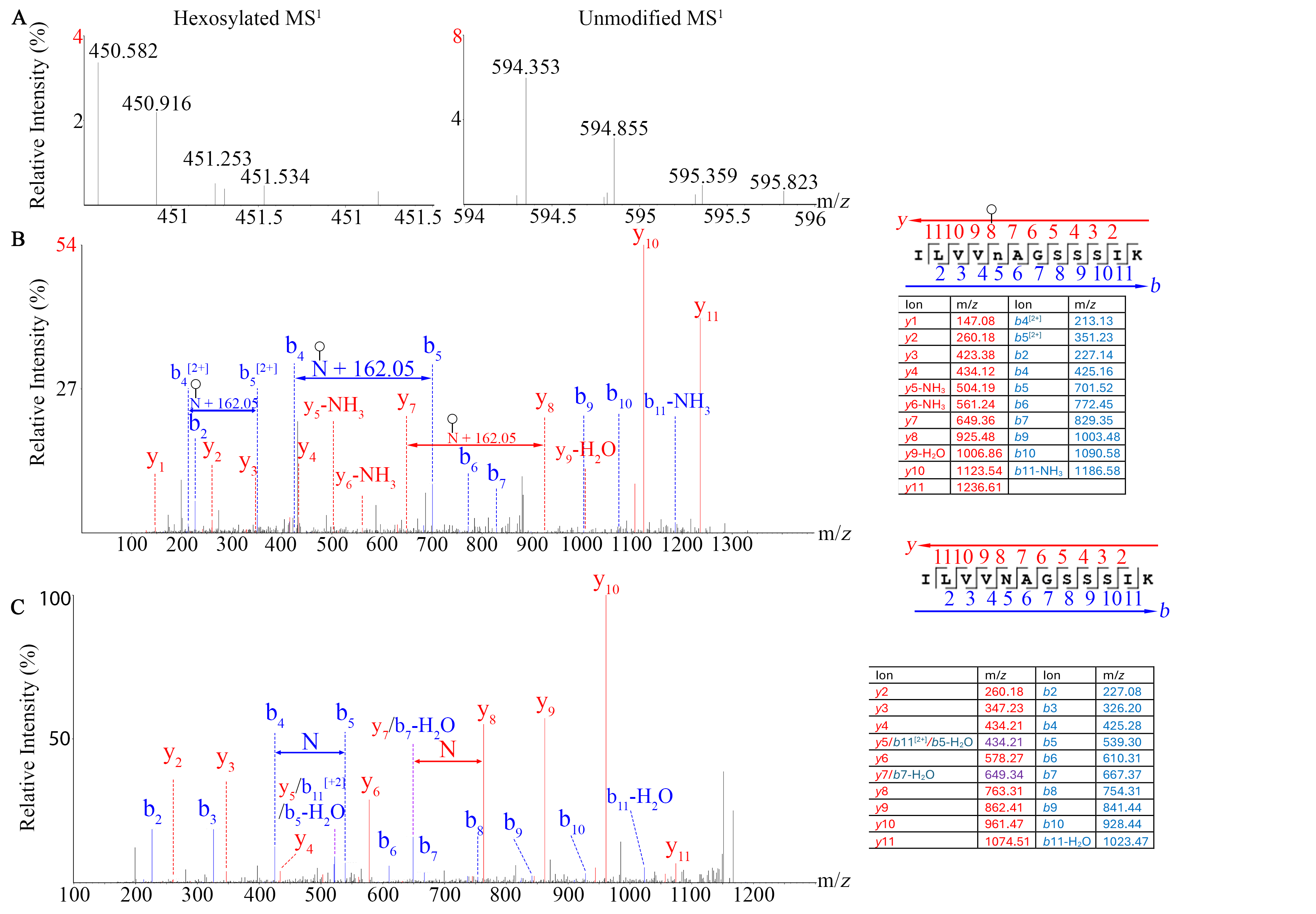

Supplement: S3 Fig — (TIF) [file pone.0329506.s003.tif]

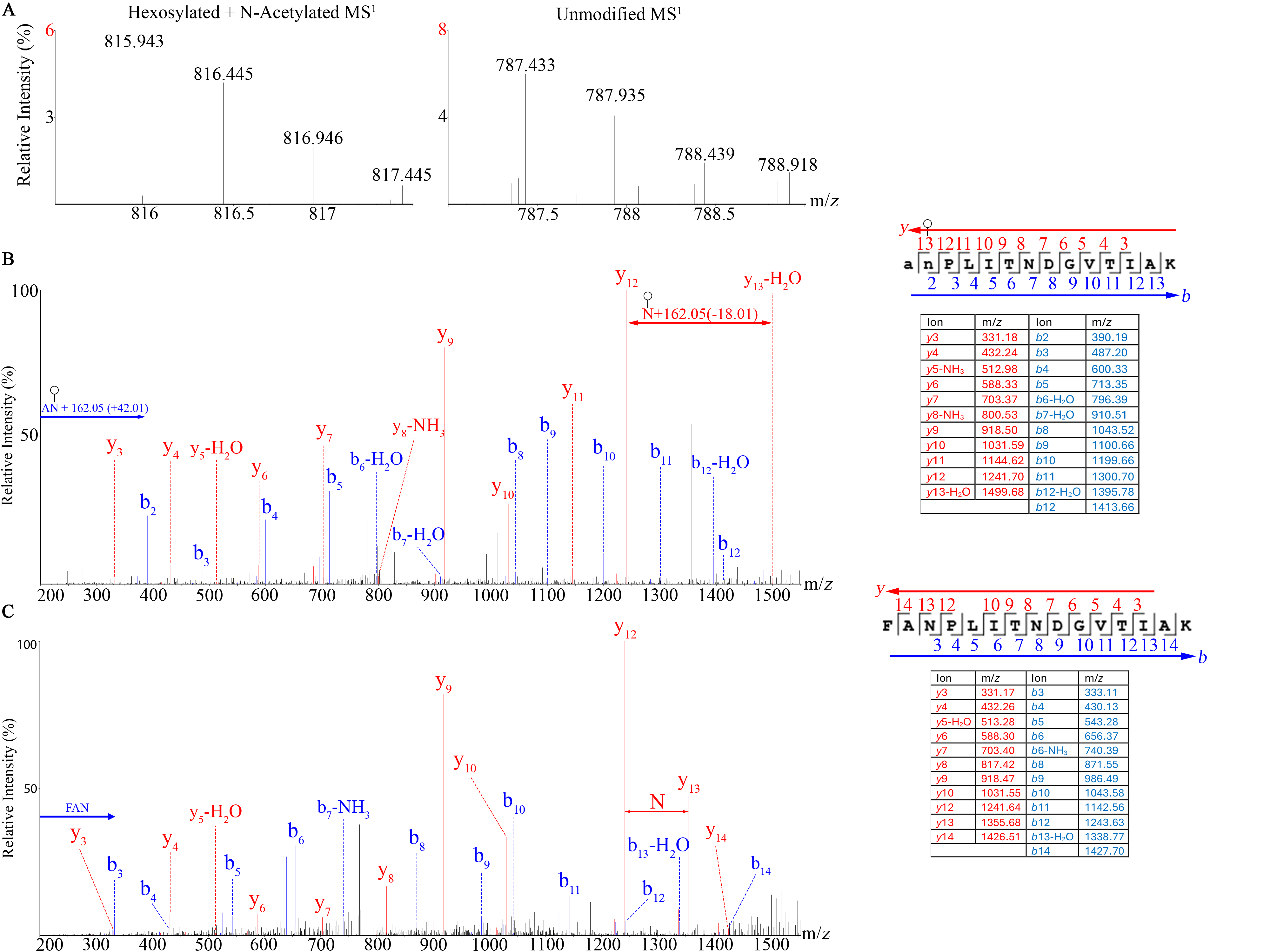

Supplement: S4 Fig — (TIF) [file pone.0329506.s004.tif]

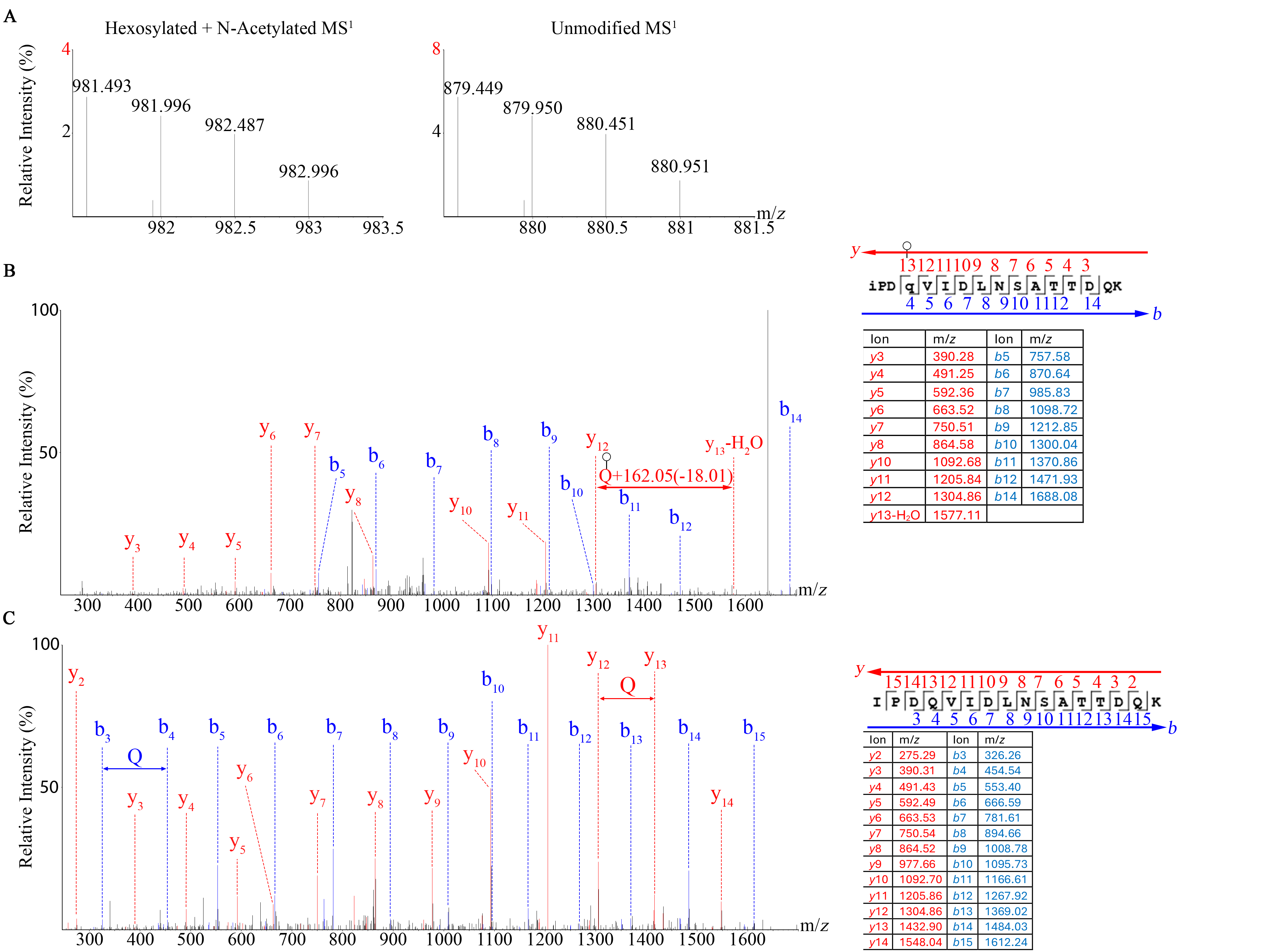

Supplement: S5 Fig — (TIF) [file pone.0329506.s005.tif]
