# Supplementary material for: Surface protein glycosylation conserved in the human pathogen Mycoplasma genitalium and retained in the synthetic organism JCVI-Syn3A
Source: PLoS One. 2025 Sep 22;20(9):e0329506. doi: 10.1371/journal.pone.0329506 (PMC12453214; doi:10.1371/journal.pone.0329506)
Supplement: S6 Fig — Green shows the locations of Ser141 (also depicted as ball-and-stick models), red shows the hydroxyl groups involved in O-bond formation. (PDF) [file pone.0329506.s006.pdf]

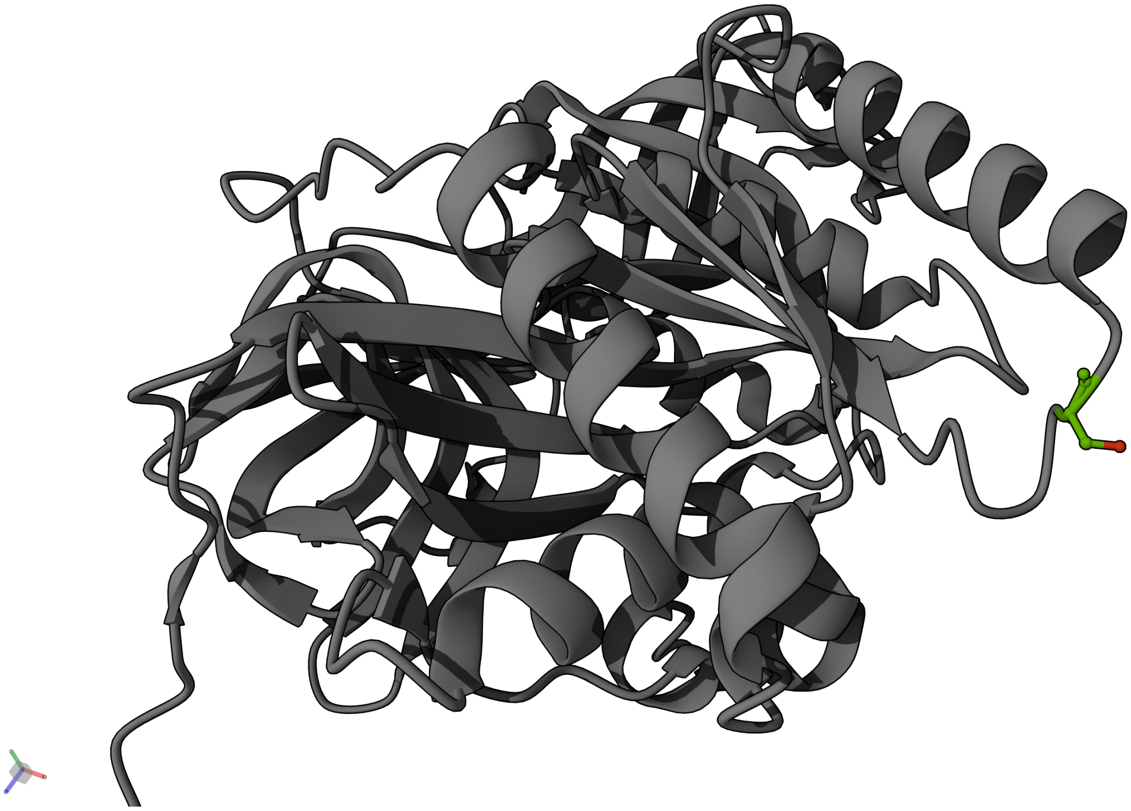

S6 Fig. Alphafold prediction of Mgen Elongation Factor-Tu. The location of Ser142 show the hydroxyl group that is O-glycosylated. Green shows the location of Ser142 (also depicted as ball-and-stick), red shows the hydroxyl groups involved in O-bond formation.
