# Supplementary material for: Surface protein glycosylation conserved in the human pathogen Mycoplasma genitalium and retained in the synthetic organism JCVI-Syn3A
Source: PLoS One. 2025 Sep 22;20(9):e0329506. doi: 10.1371/journal.pone.0329506 (PMC12453214; doi:10.1371/journal.pone.0329506)
Supplement: Attachment — Submitted filename: Response to Reviewers.pdf [file pone.0329506.s008.pdf]

July 8, 2025

Editor, PLoS ONE
1160 Battery Street
Koshland Building East, Suite 100
San Francisco, CA 94111

Dear Sir/Ms:

We thank the reviewers for their careful review of our manuscript. Please find below the detailed responses to their critiques:

Reviewer #1:
Major issues:
1) Please describe why the specific bands in Figure 1 were selected for analysis.

We have expanded the sentence starting at line 184 to say: "Two sets of bands from gel regions that have been previously shown to contain modified proteins in *M. arthritidis* and *M. pulmonis* were excised from the Coomassie-stained gel from each organism."

2) Please indicate on all figures with spectra (including supplemental figures) the relationship between the peak and the identity of the modification it represents.

We have adjusted our figure's visual design to more clearly show which peaks represent the modified amino acid.

Minor issues:
3) Line 14: Is there a reason to say "may expand" instead of "expands"?

We have removed 'may' from the sentence.

4) Line 31: "arthritidis"

We have corrected that spelling mistake and appreciate the reviewer noting it.

5) Line 37: Although the phrase "smallest genome... capable of self-replication" is often thrown around, I honestly don't know what it means. All organisms need externally-derived material to replicate; they don't "know" whether that material is provided by a living cell or something else. The distinction is not "self-replication"; the distinction is that they can be grown without other living cells.

We concur that this is a difficult phrase to word appropriately. We have changed it to "*Mycoplasma genitalium* (Mgen) is a human urogenital pathogen that has streamlined its 580-kbp genome under natural selective pressures resulting in the smallest genome of any identified organism capable of axenic culture."

6) Line 39: Biofilms are structures composed of cells as well as material they secrete or capture. Biofilms cannot be described as being secreted.

To improve the accuracy of our wording, we have changed it to "produces extracellular polymeric substances to form biofilms."

7) Figure 7 panels A-C are commonly known structures. It is unclear to me what their inclusion adds.

We concur that panels A-C were superfluous and have streamlined figure 7 to just show the hypothesized mechanism.

8) Line 292: "but" instead of "however."

We have incorporated this change.

Reviewer #2:

Minor comments:

1) Line 44: It is stated that the strain is “a highly virulent pathogen”; however, Syn3A was derived from Mycoplasma mycoides subsp. capri (Mmc), which is known to cause mild symptoms. Mmc differs from Mycoplasma mycoides subsp. mycoides small colony (Mmm), the causative agent of contagious bovine pleuropneumonia. Therefore, the term “highly” should be removed.

We appreciate the reviewer suggesting this phrasing implies Syn3A descends from Mmm, we have therefore removed ‘highly’ as suggested.

Line 65: It is recommended that the reasons for adding 3 mg/mL of maltose be briefly explained here. Although the reasons are clearly described in the Discussion section, it is not apparent when reading the Methods section alone.

We have expanded the sentence to include “3 mg/mL of maltose added to the medium to act as the required oligosaccharide substrate for mycoplasma hexosylation” to improve readability.

Lines 70–71: It may be helpful to explain the reasons for this overnight step. This step is presumed to serve the purpose of inactivating protease activity; however, the need for such prolonged treatment may be due to irreversible inhibition or the requirement for the reagent to penetrate into the cells. Including such a description would be informative for readers and would aid in better understanding.

To clarify the purpose of this step, we have changed the wording of the paragraph starting at line 71. We thank the reviewer for noting the ambiguity of this section of the methods section and hope this has clarified the purpose of the overnight equilibration step.

Line 162: The reasons for selecting bands A and B from among lots of protein bands should be provided.

We have expanded the sentence starting at line 173 to say: “Two sets of bands from gel regions that have been previously shown to contain modified proteins in *M. arthritidis* and *M. pulmonis* were excised from the Coomassie-stained gel from each organism.”

Lines 165–166 and Figure 2: In Figure 2, is it possible to add labels on the peaks corresponding to glucose and mannose?

We have added labels to show which peaks are which glycans on Figure 2, we thank the reviewer for improving the clarity of our figure.

Tables 2 and 3: Regarding the comparison between Syn3A and its parental strain Mmc,

many of the glycosylated proteins identified are different. As Syn3A is a genome-reduced strain, there are almost no additional genes compared to Mmc. Therefore, it would be expected that glycosylation detected in Syn3A should also be detectable in Mmc. The reasons for these differences should be discussed. Could this be due to the detection sensitivity or threshold settings in the MS analysis?

We appreciate the reviewer asking this astute question. We believe the variability between the modified proteins in both organisms is due to our current sample size detecting few hexosylation events. We propose that with a large enough sample, we would see more overlap between proteins conserved between the two organisms with variability in glycosylation sites, assuming our model is correct. We have added a paragraph starting at line 403 conjecturing why this is the case.

Figures 3–6

The resolution of the figures is low and the font size is too small, making it difficult to read the text in the figures. Please improve the figures by increasing both the font size and resolution. As figure readability also depends on the final published size, the font size and resolution should be adjusted accordingly.

We have increased the resolution and adjusted the fonts and visual design of our slides to be significantly more readable. We hope this is much easier to read and follow.
