# Supplementary material for: Surface protein glycosylation conserved in the human pathogen Mycoplasma genitalium and retained in the synthetic organism JCVI-Syn3A
Source: PLoS One. 2025 Sep 22;20(9):e0329506. doi: 10.1371/journal.pone.0329506 (PMC12453214; doi:10.1371/journal.pone.0329506)
Supplement: Attachment — Submitted filename: Response to Reviewers.docx [file pone.0329506.s009.docx]

July 16, 2025

Editor, PLoS ONE

1160 Battery Street

Koshland Building East, Suite 100

San Francisco, CA 94111

Dear Sir/Ms:

We thank the reviewers for their careful review of our manuscript’s revisions. Please find below the detailed responses to their critiques:

Reviewer #1:

Major issues:
In the abstract, the sentence referring to the “smallest genome… capable of self-replication” is inconsistent between the Response and the Main Text File.

There is no need to emphasize the phrase “smallest genome”, as this point is not central to the current study and may be revised as new organisms with smaller genomes are discovered in the future.
In addition, the version in the Response describes Mycoplasma genitalium as “a human urogenital pathogen that has streamlined its 580-kbp genome under natural selective pressures”. I do not agree with this use of the active voice, which incorrectly implies agency in an evolutionary process.

I suggest revising the sentence as follows:

“We report here that this surface protein hexosylation system is conserved in Mycoplasma genitalium, a human urogenital pathogen with a 580-kbp genome that can be cultured axenically.”

This manuscript can be accepted pending this final revision.

We sincerely appreciate Reviewer 1’s attention to detail and mindfulness of clear language through both rounds of peer review and have therefore incorporated their suggested sentence into the abstract.
